# Network meta-analysis of integrated traditional Chinese and Western medicine in the treatment of Sjogren’s syndrome

**DOI:** 10.3389/fphar.2024.1455969

**Published:** 2024-11-27

**Authors:** Xieli Ma, Zixia Liu, Tian Chang, Chuanhui Yao, Yuchen Yang, Biyue Shang, Jiameng Liu, Congmin Xia, Xun Gong, Quan Jiang

**Affiliations:** Department of Rheumatology, Guang’anmen Hospital, China Academy of Chinese Medical Sciences, Beijing, China

**Keywords:** Sjogren’s syndrome, Chinese patent medicine, tripterygium wilfordii polyglycosides tablets, total glucosides of paeony capsule, network meta-analysis, randomized controlled

## Abstract

**Objective:**

To evaluate efficacy and safety of traditional Chinese medicine (TCM) combined with Western medicine in treatment of Sjogren’s syndrome (SS).

**Methods:**

CNKI, WanFang, VIP, CBM, Sinomed, PubMed, Embase, and Web of Science were searched to collect randomized controlled trials (RCTs) of TCM combined with conventional western medicine (CWM) in treating SS from the time of their estalishment to May 2023. The researchers independently screened the literature and extracted data for quality evaluation. Analyses were performed using Review Manager (version 5.4) and R-4.3.1.

**Results:**

A total of 66 RCTs were included, with a sample size of 5,052, involving four kinds of TCM (total glucosides of paeony capsules, tripterygium glycosides tablet, Xinfeng capsule and Jinju Qingrun capsule) and three kinds of CWM(hydroxychloroquine sulfate, Iguratimod and glucocorticoid). The network meta-analysis results showed that IGU + HCQ + TGP ranked the highest in reducing ESR and IgG and improving the Schirmer test when the three drugs were combined. When the two drugs are combined, IGU + GC and TGT + TGP are good choices for reducing erythrocyte sedimentation rate (ESR) and Immunoglobulin G (IgG). Although TGP + HCQ vs. HCQ had the most studies, TGP combined with HCQ did not rank high in each outcome indicator. It is recommended to use TGT and XFG in decreasing ESR and IgG for a single drug. JJQR have an advantageous role in relieving xerostomia and dry eyes.

**Conclusion:**

TCM combined with CWM has a very significant effect on treating SS compared with CWM alone. According to the network meta-analysis, the best intervention measures of different TCMs for different outcome indicators were obtained.

**Systematic Review Registration:**

[https://www.crd.york.ac.uk/prospero/], identifier [CRD42023451845].

## 1 Introduction

Sjogren’s syndrome (SS) is a systemic autoimmune disease mainly involving exocrine glands. Its pathological feature is lymphocyte and plasma cell infiltration (Ramos-Casals et al., 2012). The main clinical manifestations are dry mouth and dry eyes, and organ involvement can also occur, such as the digestive system, lung, kidney, etc (Mariette and Criswell, 2018). In addition, about 5%–10% of SS patients are associated with lymphoma (Beydon et al., 2024). The prevalence of the elderly in China is as high as 3.00%–4.00%; The prevalence of SS in Europe is about 0.23% (Brito-Zerón et al., 2016). The pathogenesis of PSS is complex, involving genetic, environmental factors and abnormal activation of the immune system. The activation of B cells and T cells plays an important role in maintaining and exacerbating the inflammatory response (Baldini et al., 2024).

At present, the treatment options for SS are minimal, mainly immunosuppressive drugs and biological agents. In addition to symptomatic treatment to alleviate symptoms, there is no clear indication for drugs (Seror et al., 2021). Traditional Chinese medicine (TCM) has the advantage of multiple links, pathways, and targets, excelling in treating SS from a holistic perspective (Wang et al., 2024). With the development of TCM, the clinical application of TCM or the combination of TCM and Western medicine in treating SS is becoming increasingly widespread. For example, some studies have found that traditional Chinese medicine exerts therapeutic effects on SS mice and NOD mice by inhibiting inflammatory responses (Li et al., 2020). Other research indicates that total glycosides of white peony can improve the pathological damage of the submandibular glands in SS mice, possibly playing a therapeutic role in SS through the immune balance between Th17 and Treg mediated by RORγt/FoxP3 (Wu et al., 2021). There needs to be more comparison of the efficacy of different Chinese patents and Western medicines in treating SS. Network meta-analysis can quantitatively synthesize the results of multiple independent studies to enhance the strength and accuracy of evidence. In addition, in the absence of direct comparison, it can indirectly compare the effects of different interventions, so as to provide scientific basis for clinical practice and provide broader extrapolation (Higgins and Welton, 2015). Therefore, this study aims to conduct a network meta-analysis of Chinese and Western medicine interventions for Sjogren’s syndrome and to explore the efficacy and safety ranking of the current treatment of Sjogren’s syndrome to guide the best clinical treatment measures. The composition table of the traditional Chinese medicine is shown in Table 1.

**TABLE 1:** Incorporate traditional Chinese medicine components.

Acronym	Name	Composition
TGT	Tripterygium glycosides tablet	Tripterygium glycosides
TGP	Total glucosides of paeony capsules	Total glucosides of paeony
XFC	Xinfeng capsule	Astragalus membranaceus, semen coicis, thunder god vine, centipede
JJQR	Jinju Qingrun capsule	Ginseng, radix scrophulariae, ophiopogon japonicus, danshen, honeysuckle, loofah, radix paeonies Rubra, wild chrysanthemum, pangolin

## 2 Methods

### 2.1 Literature search strategy

The study has been registered in the International Registry of Prospective Systematic Reviews (PROSPERO), and the registration number is CRD42023451845. Citations from the time of their estalishment to May 2023 were searched for in CNKI, Wanfang, VIP, CBM, Sinomed, PubMed, Embase, and Web of Science databases. The screening criteria were randomized controlled trials of TCM and CWM to treat SS. The included herbs included total glucosides of Paeony capsules, Tripterygium wilfordii polyglycosides tablets, Xinfeng capsules and Jinju Qingrun capsule. The included western drugs included hydroxychloroquine sulfate, methotrexate, iguratimod, leflunomide, methylprednisolone, prednisone. A literature search was conducted independently by two researchers, “Tripterygium wilfordii polyglycosides,” “total glucosides of paeony capsules,” “Pafflin capsules,” “Xinfeng capsules,” “Conventional western medicine,” “methotrexate,” “hydroxychloroquine,” “leflunomide,” “Iguratid,” “hormone,” “methylprednisolone,” “prednisone,” “hydrocortisone” and “Sjogren’s syndrome” were used for literature search in the database. A comprehensive search strategy is shown in Supplementary Table S1. Two researchers independently conducted the literature search. Weights were selected, and literature was screened based on title, abstract, and full-text reading for final inclusion.

### 2.2 Inclusion criteria

(1) The study type belonged to those above randomized controlled trials of Chinese patent medicine and Western medicine. it includes the treatment of SS with traditional Chinese medicine alone, Western medicine alone, or a combination of both, without any language restrictions. (2) The subjects should meet the classification criteria for primary Sjogren syndrome set by the 2002 American European Consensus Group (AECG) or the 2016 American College of Rheumatology/European Alliance against Rheumatism (ACR/EULAR) (Vitali et al., 2002; Shiboski et al., 2017). There were no special requirements for age, region, race, or gender.

### 2.3 Exclusion criteria

(1) Articles published were animal or cell experiments, academic conferences, reviews, or non-randomized controlled trials; (2) Duplicate published academic literature; (3) Interventions did not meet the requirements of the literature; (4) Literature for which complete data could not be obtained after contacting the authors.

### 2.4 Study extractions and quality assessment

Two researchers independently screened the literature according to the inclusion and exclusion criteria, extracted the data using a pre-prepared Excel sheet, and assessed the risk of bias in the included literature. In the event of a disagreement, we will work towards a resolution through discussion or seek the assistance of a third-party mediator. The quality of the literature was assessed according to the bias risk tool of Cochrane assessment manual 5.1.0, and RevMan 5.4 software was used to draw the risk of bias map. The quality assessment criteria were as follows: random sequence generation of literature quality assessment; Assign hidden methods; Whether investigators, participants, and outcome assessors were blinded; The integrity of outcome data; Selective reporting of results; There were no other biases. The publication bias was evaluated by low, unclear, and high risks, and two researchers cross-checked the results.

### 2.5 Outcome measures

Based on the consensus experience of clinical experts and the pooled outcome measures in the RCT, we selected: erythrocyte sedimentation rate (ESR), immunoglobulin G (IgG) level, Schirmer test, salivary flow rate, total response rate and adverse events as outcome measures. The total response rate was calculated as follows: (number of cured patients + number of improved patients)/total number of patients 100%. When the patient’s clinical symptoms and objective indicators disappear, the patient returns to normal. The patient had clinical symptoms and objective indicators, and the condition was considered to have improved. If the clinical symptoms and objective indicators were unchanged or aggravated, the patient was determined as having ineffective efficacy status.

### 2.6 Statistical analysis

The R-4.3.1 package “netmeta” was utilized to analyze the literature. Statistical heterogeneity was calculated using the I^2^ statistic, which describes the percentage of total variation across studies due to heterogeneity rather than chance. We defined an I^2^ greater than 50% as indicating substantial heterogeneity, in which case a random-effects model was used. Furthermore, given the common differences in population characteristics and study designs across studies, we ultimately reported only the results from the random-effects model. Odds ratio (OR) was used for binary variables, mean difference (MD) for continuous variables, and a 95% confidence interval (CI) was calculated. The potential scale reduction factor (PSRF) indicated stability; the closer the value is to 1, the more stable the result. The “mtc. model ()” function established the consistency model. The “gelman. plot ()” function drew the convergence diagnosis and trajectory density maps. The “mtc. network ()” function drew the n network evidence diagram. The “forest ()” function was used to draw the forest plot of direct comparison between different interventions and conventional Western medicine. The “mtc. run ()” function was used to calculate the effect size of each intervention pairwise comparison and output the league table. The “rank. probability ()” function was used to calculate the surface under the cumulative ranking curve (SUCRA) of each intervention and draw the cumulative probability ranking graph. The intervention was considered more effective based on a higher SUCRA value (Yi et al., 2015).

## 3 Results

### 3.1 Literature search results

**FIGURE 1:**
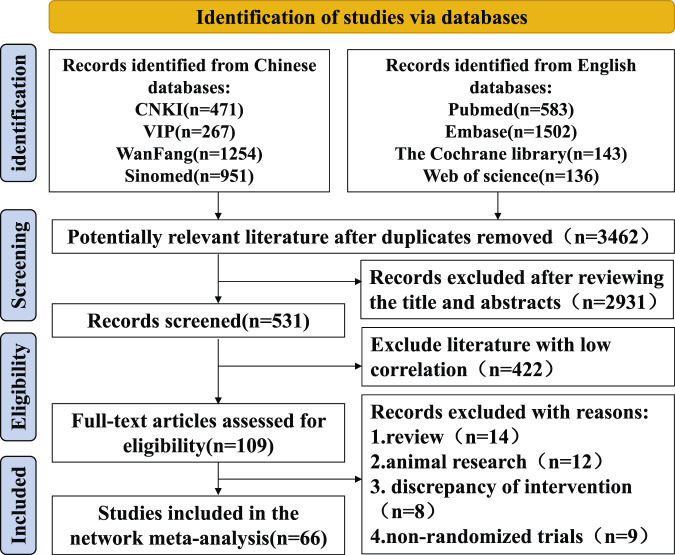
PRISMA flow diagram.

Five thousand three hundred seven articles were retrieved, and 3,462 remained after excluding duplicate articles using NoteExpress. A total of 2,931 articles were excluded after scanning titles and abstracts strictly according to the inclusion and exclusion criteria, and 66 articles were finally included after reading the full text (Chen and Chen, 2017; Chen et al., 2022; Chu, 2021; Ding et al., 2022; Fan et al., 2015; Feng et al., 2021; Gan et al., 2022; Gao, 2021; Gu, 2020; Gu, 2022; Guo et al., 2012; He, 2010; Ji and Cheng, 2019; Jia, 2020; Jiang et al., 2016; Jiang et al., 2014; Ju et al., 2022; Li et al., 2018; Li, 2019; Li et al., 2020; Li et al., 2016; Li and Li, 2022; Liu, 2022; Liu and Yan, 2020; Liu et al., 2023; Liu J. et al., 2022; Lu et al., 2019; Lu and Zhang, 2021; Luo et al., 2019; Luo et al., 2018; Ma, 2012; Ma et al., 2021; Meng et al., 2023; Rao et al., 2022; Shao, 2016; Shi and Kong, 2023; Tang et al., 2020; Wang et al., 2013; Wang et al., 2014; Wang, 2019; Wang, 2017; Wang, 2018; Wang et al., 2019; Wang and Wang, 2020; Wu et al., 2017; Xia et al., 2017; Xie et al., 2020; Xu et al., 2017; Xu et al., 2022; Yang et al., 2011; Ye et al., 2019; Yin, 2011; Yu, 2020; Zhang and Shen, 2019; Zhang et al., 2011; Zhang et al., 2009; Zhang, 2015; Zhang, 2019; Zhang, 2021; Zhao, 2013; Zhao, 2018; Zhao, 2020; Zhao, 2023; Zhao, 2019a; Zhao, 2019b; Zhu et al., 2016). The literature screening process was as follows Figure 1. A total of 5,052 cases were enrolled, including 2,529 cases in the experimental group and 2,523 cases in the control group. The sample size of a single study ranged from 29 to 200 cases, covering four kinds of Chinese patent medicine, including Tripterygium wilfordii polyglycosides tablets, total glucosides of paeony capsules, Xinfeng capsules, and Jinjuqingruncapsule.

### 3.2 Description of included trials

The essential characteristics of the included literature are shown in Table 2.

**TABLE 2:** Basic features included in the study.

Study	Total sample size	Age	Course of disease/year	Course/month	Intervention	Outcome indicator
Wang et al. (2013)	64	55.26 ± 12.38vs55.10 ± 6.50	6.21 ± 4.43vs7.80 ± 3.71	3	XFC/HCQ	④⑤
Zhu et al. (2016)	58	52 ± 21vs53 ± 24	9 ± 5vs14 ± 10	3	XFC/HCQ	①⑤⑥
Ma (2012)	44	50 ± 7.14vs49 ± 8.19	19 ± 6.46vs17 ± 8.32	3	TGT/HCQ	①②⑥
Guo et al. (2012)	29	51.9 ± 7.1vs52.3 ± 7.2	3.9 ± 5.7vs3.4 ± 5.8	3	TGT/HCQ	①②⑥
Ma et al. (2021)	60	50.7 ± 11.4vs51.5 ± 10.7	3.5 ± 7.0vs3.1 ± 6.7	3	TGT/HCQ	①②⑤⑥
Zhao (2020)	50	45.3 ± 2.8vs45.7 ± 2.8	4.12 ± 0.23vs4.01 ± 0.20	3	IGU/HCQ	①②⑤⑥
Fan et al. (2015)	40	56.25 ± 11.40vs55.16 ± 12.22	6.22 ± 4.30vs6.18 ± 3.26	1	XFC/TGP	①②⑤
Wang et al. (2014)	60	49.10 ± 7.11vs48.60 ± 9.20	6.21 ± 4.76vs6.28 ± 5.72	3	XFC/TGP	⑤
Shao (2016)	40	48.21 ± 6.22vs47.93 ± 6.42	6.13 ± 4.34vs6.64 ± 5.01	3	XFC/TGP	①②④⑤
Yang et al. (2011)	38	55.95 ± 12.52vs54.58 ± 12.54	6.39 ± 4.24vs6.03 ± 4.71	3	XFC/TGP	①②⑤
Zhang et al. (2011)	57	37.00 ± 12vs38 ± 13.00	5 ± 9vs6±8	3	JJQR/HCQ	①②③④⑤⑥
Zhang et al. (2009)	95	39.95 ± 11.58vs41.33 ± 12.59	5.20 ± 9.35vs5.60 ± 7.63	3	JJQR/GC	①②③④⑤⑥
Liu and Yan (2020)	76	47.28 ± 7.34vs47.63 ± 6.92	4.59 ± 1.57vs4.73 ± 1.65	3	TGP + TGT/TGT	①⑤
Ye et al. (2019)	74	45.73 ± 2.45vs45.64 ± 2.38	—	3	TGP + TGT/TGT	⑤
Wu et al. (2017)	60	46.29 ± 6.31vs47.92 ± 7.05	5.02 ± 3.12vs4.93 ± 3.41	3	TGP + TGT/TGT	①⑤
Wang (2017)	98	49.7 ± 5.8vs50.1 ± 5.6	-	3	TGP + TGT/TGT	④⑤⑥
Zhao (2019a)	84	51.52 ± 6.22vs50.53 ± 6.24	6.51 ± 1.54vs5.52 ± 1.56	3	TGP + TGT/TGT	⑤⑥
Gan et al. (2022)	114	44.0 ± 3.6vs44.2 ± 3.9	1.30 ± 0.33vs1.27 ± 0.36	3	TGT + TGP/TGP	⑤⑥
Lu and Zhang (2021)	96	45.52 ± 7.48vs44.24 ± 8.32	3.43 ± 0.26vs3.42 ± 0.25	3	IGU + HCQ/HCQ	①②⑤⑥
Ji and Cheng (2019)	82	43.70 ± 5.00vs44.30 ± 5.60	3.20 ± 0.90vs3.00 ± 1.00	3	IGU + HCQ/HCQ	①②③④⑤
Zhao (2018)	200	49.8 ± 5.4vs50.1 ± 5.6	5.4 ± 0.6vs5.5 ± 0.5	3	TGP + HCQ/HCQ	④⑤⑥
Chen and Chen (2017)	62	53.02 ± 5.12vs55.03 ± 4.92	5.11 ± 1.07vs4.22 ± 1.13	2	TGP + HCQ/HCQ	⑤
Gao (2021)	100	53.28 ± 5.06vs54.35 ± 5.74	3.43 ± 1.12vs3.59 ± 0.67	3	TGP + HCQ/HCQ	②④⑤
Shi and Kong (2023)	80	49.45 ± 6.81vs49.68 ± 5.62	3.72 ± 1.26vs4.03 ± 1.48	6	TGP + HCQ/HCQ	①③④⑤⑥
He (2010)	48	35 ± 12	1.33 ± 1.17	3	TGP + HCQ/HCQ	①②③④⑤⑥
Li et al. (2016)	90	54.7 ± 23.1	1.25 ± 1.08	2	TGP + HCQ/HCQ	⑤
Wang (2019)	60	50.22 ± 14.76vs52.07 ± 15.85	—	3	TGP + HCQ/HCQ	④⑤⑥
Tang et al. (2020)	66	56.36 ± 8.99vs56.42 ± 10.41	3.58 ± 1.21vs3.12 ± 1.11	3	TGP + HCQ/HCQ	①②⑤
Zhang (2015)	60	56.10 ± 7.54vs53.00 ± 5.09	5.07 ± 2.89vs3.80 ± 1.97	6	TGP + HCQ/HCQ	①⑤⑥
Yin (2011)	81	48 ± 13	2.25 ± 1.75	3	TGP + HCQ/HCQ	①②③④
Liu M. et al. (2022)	62	47.03 ± 6.57vs47.35 ± 6.83	—	3	TGP + HCQ/HCQ	①②⑤
Lu et al. (2019)	100	51.88 ± 10.24vs52.40 ± 9.18	7.05 ± 4.16vs7.13 ± 3.20	3	TGP + HCQ/HCQ	①②⑤⑥
Zhao (2023)	59	52.45 ± 8.95vs52.52 ± 8.93	7.10 ± 2.15vs7.12 ± 2.18	3	TGP + HCQ/HCQ	①②④⑤
Xu et al. (2022)	100	52.18 ± 4.67vs51.97 ± 4.32	4.28 ± 0.95vs4.05 ± 0.87	2	TGP + HCQ/HCQ	③⑤⑥
Chu (2021)	56	54.31 ± 3.29vs54.23 ± 3.27	4.19 ± 0.43vs4.21 ± 0.40	3	TGP + HCQ/HCQ	②⑤
Li (2019)	46	40.72 ± 5.59vs40.24 ± 5.38	—	6	IGU + TGP/IGU	①②
Zhao and Zhao (2013)	84	42.9 ± 11.6vs44.5 ± 11.6vs44.1 ± 10.8	1.09 ± 0.45vs1.05 ± 0.50vs0.84 ± 0.61	6	TGP + HCQ/HCQ/TGP	①②③④⑤
Feng et al. (2021)	194	45.02 ± 13.47vs45.36 ± 13.08	6.91 ± 2.08vs7.13 ± 2.04	3	HCQ + GC/GC	①②⑤⑥
Zhang (2019)	120	49.43 ± 3.74	—	3	IGU + GC/HCQ + GC	③⑤
Zhang and Shen (2019)	86	40.35 ± 9.41vs41.03 ± 10.01	2.31 ± 0.61vs2.20 ± 0.52	3	IGU + GC/HCQ + GC	①②③④⑤⑥
Xu et al. (2017)	94	44.5 ± 13.2vs45.3 ± 13.1	6.12 ± 1.82vs5.96 ± 1.73	3	IGU + GC/HCQ + GC	①②③④⑤⑥
Zhao (2019b)	82	55.51 ± 6.52vs54.52 ± 6.54	4.53 ± 0.84vs4.52 ± 6.54	3	IGU + GC/HCQ + GC	①②⑤⑥
Yu (2020)	76	41.18 ± 3.36vs 41.14 ± 3.39	5.15 ± 0.62vs5.12 ± 0.66	3	IGU + GC/HCQ + GC	①②
Gu (2020)	80	66.72 ± 4.34vs 66.51 ± 4.23	4.28 ± 1.40vs4.36 ± 1.35	3	IGU + GC/HCQ + GC	②⑤⑥
Jiang et al. (2016)	60	45.13 ± 12.11vs46.33 ± 13.74	6.01 ± 2.34vs4.90 ± 2.67	3	IGU + GC/HCQ + GC	①②
Gu (2022)	84	40.97 ± 10.24vs41.56 ± 10.21	2.42 ± 0.71vs2.48 ± 0.72	3	IGU + GC/HCQ + GC	②⑤⑥
Xia et al. (2017)	100	42.13 ± 9.97vs42.08 ± 9.65	—	3	IGU + GC/HCQ + GC	①②
Wang and Wang (2020)	60	55.29 ± 5.62vs54.32 ± 5.45	—	3	IGU + GC/HCQ + GC	⑤
Wang (2018)	76	48.13 ± 7.21vs48.22 ± 7.18	6.19 ± 1.37vs6.18 ± 1.36	3	IGU + GC/HCQ + GC	①②⑤
Luo et al. (2018)	80	43.6 ± 10.5vs 45.2 ± 12.9	6∼7vs6∼8	3	IGU + GC/HCQ + GC	①②⑤⑥
Li and Li (2022)	136	72.71 ± 12.59vs72.65 ± 12.62	15.38 ± 8.05vs15.34 ± 8.02	3	IGU + GC/HCQ + GC	②⑤⑥
Liu et al. (2023)	97	45.23 ± 7.52vs44.86 ± 7.24	3.86 ± 1.01vs4.08 ± 1.16	2	IGU + GC/HCQ + TGP + GC	②⑤⑥
Liu (2022)	80	44.05 ± 8.82vs43.68 ± 8.75	2.37 ± 0.61vs2.25 ± 0.58	3	IGU + GC/HCQ + TGP + GC	①②③④⑤⑥
Ding et al. (2022)	40	66.15 ± 3.71vs 66.31 ± 3.98	2.93 ± 0.79vs2.85 ± 0.79	3	IGU + HCQ + GC/HCQ + GC	①②③④⑥
Jiang et al. (2014)	50	29.3 ± 9.7vs32.5 ± 11.5	0.67∼2.67vs0.83∼3.00	3	IGU + HCQ + GC/HCQ + GC	②③⑤⑥
Li et al. (2020)	46	46.29 ± 1.24vs 46.38 ± 1.37	—	3	IGU + HCQ + GC/HCQ + GC	①②④⑤⑥
Meng et al. (2023)	60	56.5 ± 15.6vs58.1 ± 16.9	3.20 ± 3.30vs3.70 ± 2.80	3	IGU + HCQ + GC/HCQ + GC	②⑥
Rao et al. (2022)	86	51.8 ± 10.3vs50.1 ± 9.9	2.0 ± 0.5vs2.2 ± 0.6	3	IGU + HCQ + GC/HCQ + GC	①②③⑤
Jia (2020)	86	50.47 ± 9.11vs 50.47 ± 9.11	4.51 ± 1.46vs4.51 ± 1.46	3	IGU + HCQ + GC/HCQ + GC	①②⑥
Luo et al. (2019)	73	49.7 ± 12.3	—	6	IGU + HCQ + GC/HCQ + GC	①②⑥
Li et al. (2018)	68	40.05 ± 3.16vs 40.02 ± 3.15	3.43 ± 0.26vs3.51 ± 0.26	3	IGU + HCQ + GC/HCQ + GC	⑤⑥
Wang et al. (2019)	64	66.8 ± 7.7vs65.3 ± 8.2	0.5∼10.83vs0.67∼10.00	3	IGU + HCQ + TGP/HCQ + TGP	①②③④⑤⑥
Zhang (2021)	70	66.3 ± 7.3vs 65.4 ± 7.1	3.37 ± 0.59vs3.26 ± 0.57	6	IGU + HCQ + TGP/HCQ + TGP	①③④⑤
Xie et al. (2020)	76	57.3 ± 7.92vs56.8 ± 8.44	0.73 ± 0.49vs0.79 ± 0.41	6	IGU + HCQ + TGP/HCQ + TGP	①②③⑤⑥
Chen et al. (2022)	125	68.50 ± 3.05vs 68.02 ± 3.02	—	3	IGU + HCQ + TGP/HCQ + TGP	①②
Ju et al. (2022)	60	52.34 ± 3.09vs 52.26 ± 3.02	2.50 ± 0.35vs2.45 ± 0.32	3	TGT + HCQ + GC/HCQ + GC	②⑤⑥

Note: TGP, total glucosides of paeony capsule; TGT, tripterygium glycosides tablets; XFC, xinfeng capsule; JJQR, jinju qingrun capsule; HCQ, hydroxychloroquine sulfate tablets; IGU, iguratimod film; GC, hormones; ①ESR; ②IgG; ③Schirmer trial; ④ Salivary flow rate; ⑤Total effective rate; ⑥ Adverse events.

### 3.3 Risk of bias

**FIGURE 2:**
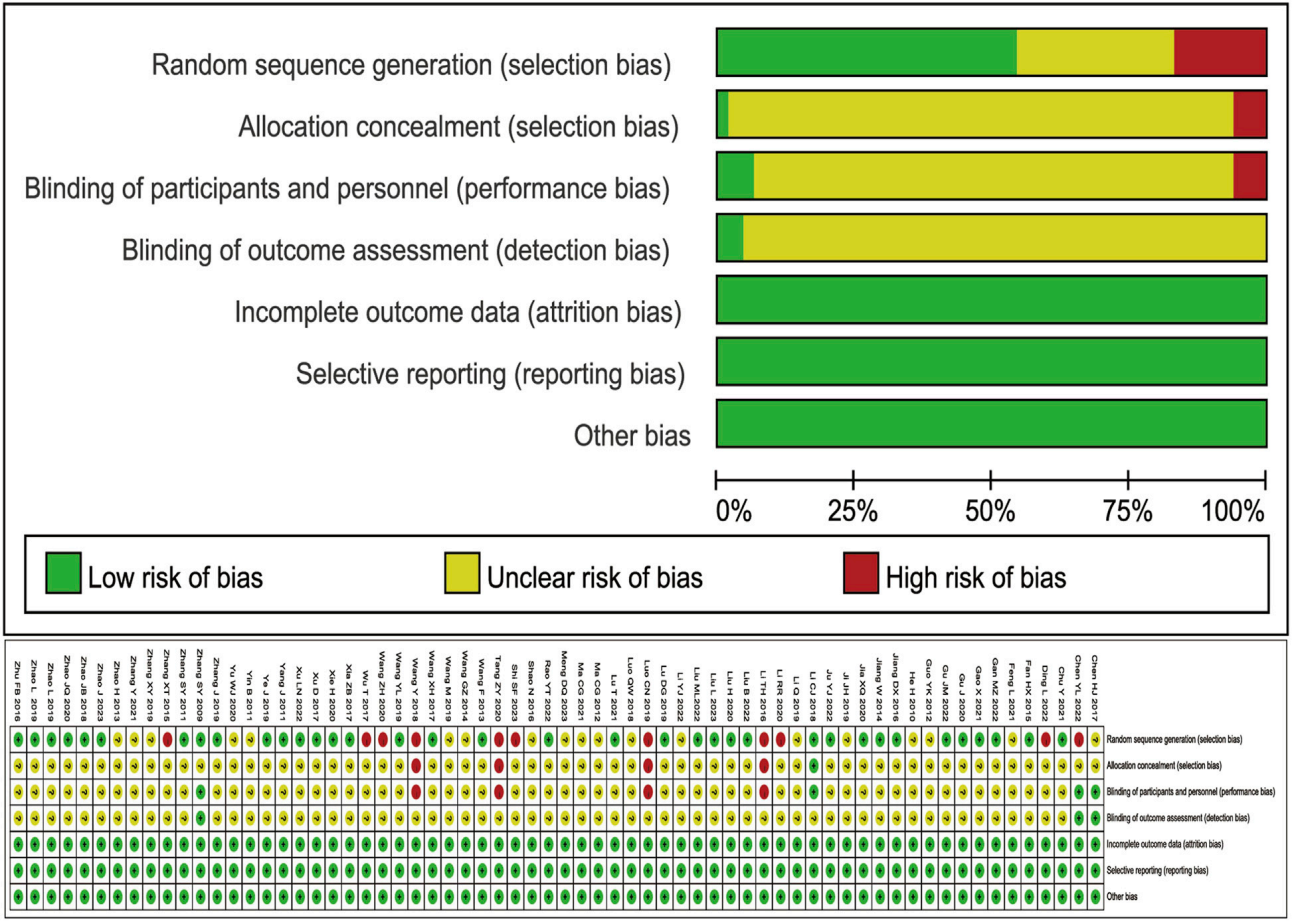
Literature quality evaluation chart.

Of the 66 included RCTS, 29 items (Chen et al., 2022; Chu, 2021; Fan et al., 2015; Gan et al., 2022; Gao, 2021; Gu, 2020; Gu, 2022; Jia, 2020; Jiang et al., 2016; Ju et al., 2022; Li et al., 2018; Liu and Yan, 2020; Liu et al., 2023; Liu M. et al., 2022; Lu et al., 2019; Lu and Zhang, 2021; Wang et al., 2013; Wang, 2017; Wang et al., 2019; Xie et al., 2020; Xu et al., 2017; Xu et al., 2022; Yang et al., 2011; Zhang and Shen, 2019; Zhao, 2018; Zhao, 2020; Zhao, 2019a; Zhao, 2019b; Zhu et al., 2016) using a random number table method, 3 items (Jiang et al., 2014; Zhang et al., 2011; Zhang et al., 2009) were SAS/SPSS statistical software, 5 items (Liu, 2022; Rao et al., 2022; Xia et al., 2017; Ye et al., 2019; Zhao, 2023) were lottery method, and the risk of bias was rated as low risk, 6 items (Li and Long, 2020; Luo et al., 2019; Shi and Kong, 2023; Tang et al., 2020; Wang, 2018; Wang and Wang, 2020) were grouped by different treatment regimens, 4 items (Ding et al., 2022; Li et al., 2016; Wu et al., 2017; Zhang, 2015) were grouped by admission or visit order. The risk of bias was rated as high, and the rest of the studies only mentioned the word “random,” and the risk of bias was rated as unclear. One article (Li et al., 2018) mentioned single-blind and allocation concealment, and the risk of bias was rated as low. All studies did not mention loss to follow-up/dropout reports, and there was no attrition bias. All studies had complete outcome data without selective reporting bias. Whether other biases are present is unclear. The quality evaluation of the included literature is shown in Figure 2.

### 3.4 ESR

#### 3.4.1 Evidence network and network meta-analysis

**FIGURE 3:**
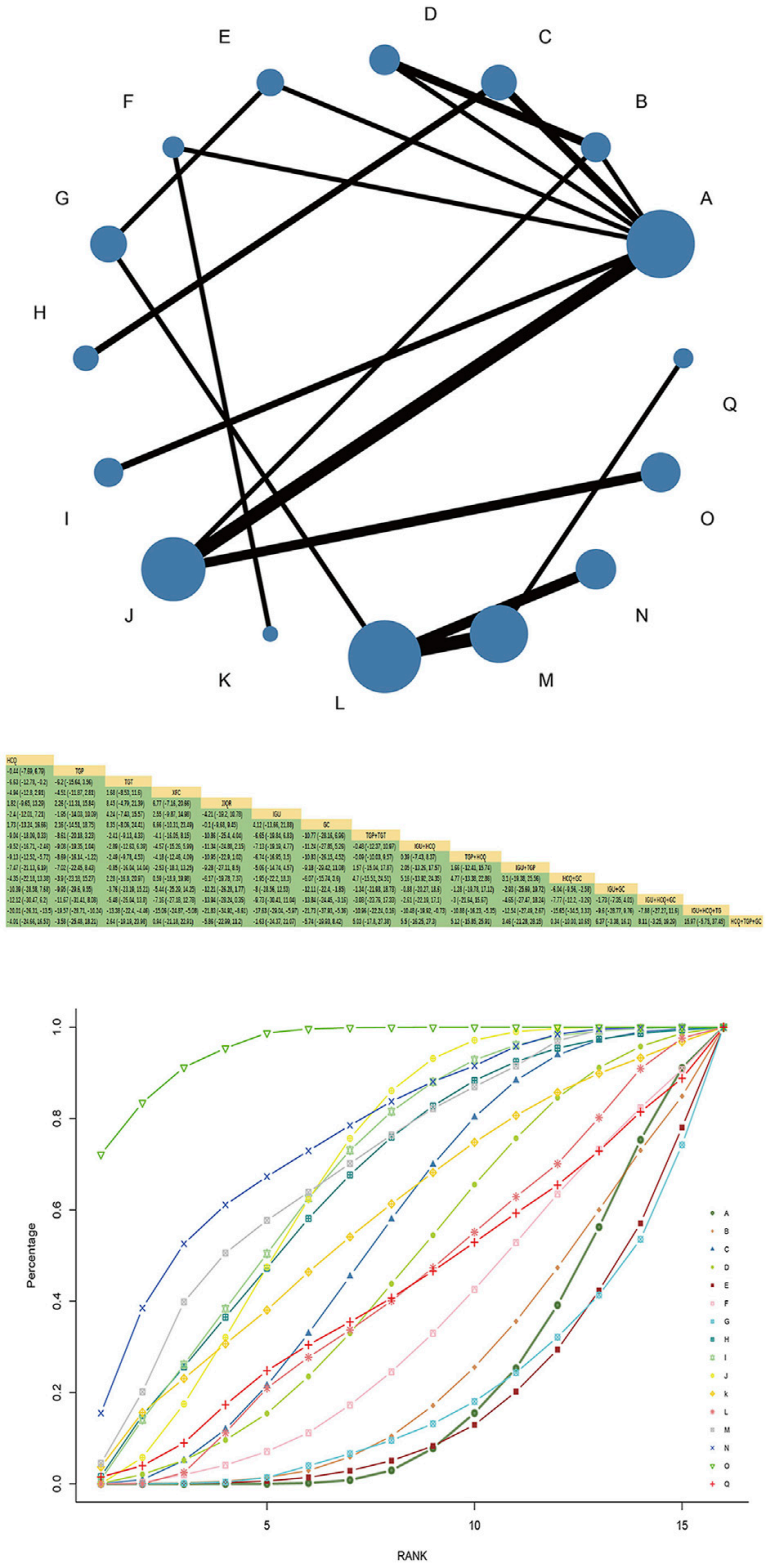
ESR network diagram, league diagram, SUCRA diagram. Note: A: HCQ; B: TGP; C: TGT; D: XFC; E: JJQR; F: IGU; G: GC; H: TGP + TGT; I: IGU + HCQ; J: TGP + HCQ; K: IGU + TGP; L: HCQ + GC; M: IGU + GC; N: IGU + HCQ + GC; O: IGU + HCQ + TGP; P: TGT + HCQ + GC; Q: HCQ + TGP + GC; In the evidence network drawn by different outcome indicators, the node size represents the study’s sample size, and the node connection’s thickness represents the number of included studies, the same as below.

Forty-three RCTs reported ESR, involving 16 interventions, four kinds of Chinese patent medicine, 3,059 patients, and two closed loops. HCQ + TGPvsHCQ had the largest comparison with the thickest line segment and greater sample size. HCQ + GC had the largest nodes and sample size studied, with the most in direct comparison with IGU + GC.The results of the network meta-analysis showed that TGT had better efficacy than HCQ [MD = −6.63, 95%CI= (−12.78, −0.2)], TGP, XFC, JJQR had no significant difference compared with HCQ and IGU (*p* > 0.05). TGP combined with HCQ was superior to HCQ alone [MD = −9.13, 95%CI= (−12.52, −5.72)](Figure 3). The SUCRA ranking in the network graph of each outcome indicator is shown in Supplementary Table S2.

#### 3.4.2 SUCRA probability ranking

SUCRA probability values are ranked as:IGU + HCQ + TGP(SUCRA = 96.0)>IGU + HCQ + GC(SUCRA = 76.4)>IGU + GC(SUCRA = 69.5)>IGU + HCQ (SUCRA = 67.9)>TGP + HCQ (SUCRA = 67.6)>TGP + TGT (SUCRA = 65.5)>IGU + TGP(SUCRA = 57.4)>TGT (SUCRA = 53.7)>XFC(SUCRA = 46.4)>HCQ + GC(SUCRA = 42.8)>HCQ + TGP + GC(SUCRA = 42.2)>IGU(SUCRA = 33.6)>TGP(SUCRA = 24.2)>HCQ (SUCRA = 20.9)>GC(SUCRA = 18.7)>JJQR (SUCRA = 17.3).

### 3.5 IgG

#### 3.5.1 Evidence network and network meta-analysis

**FIGURE 4:**
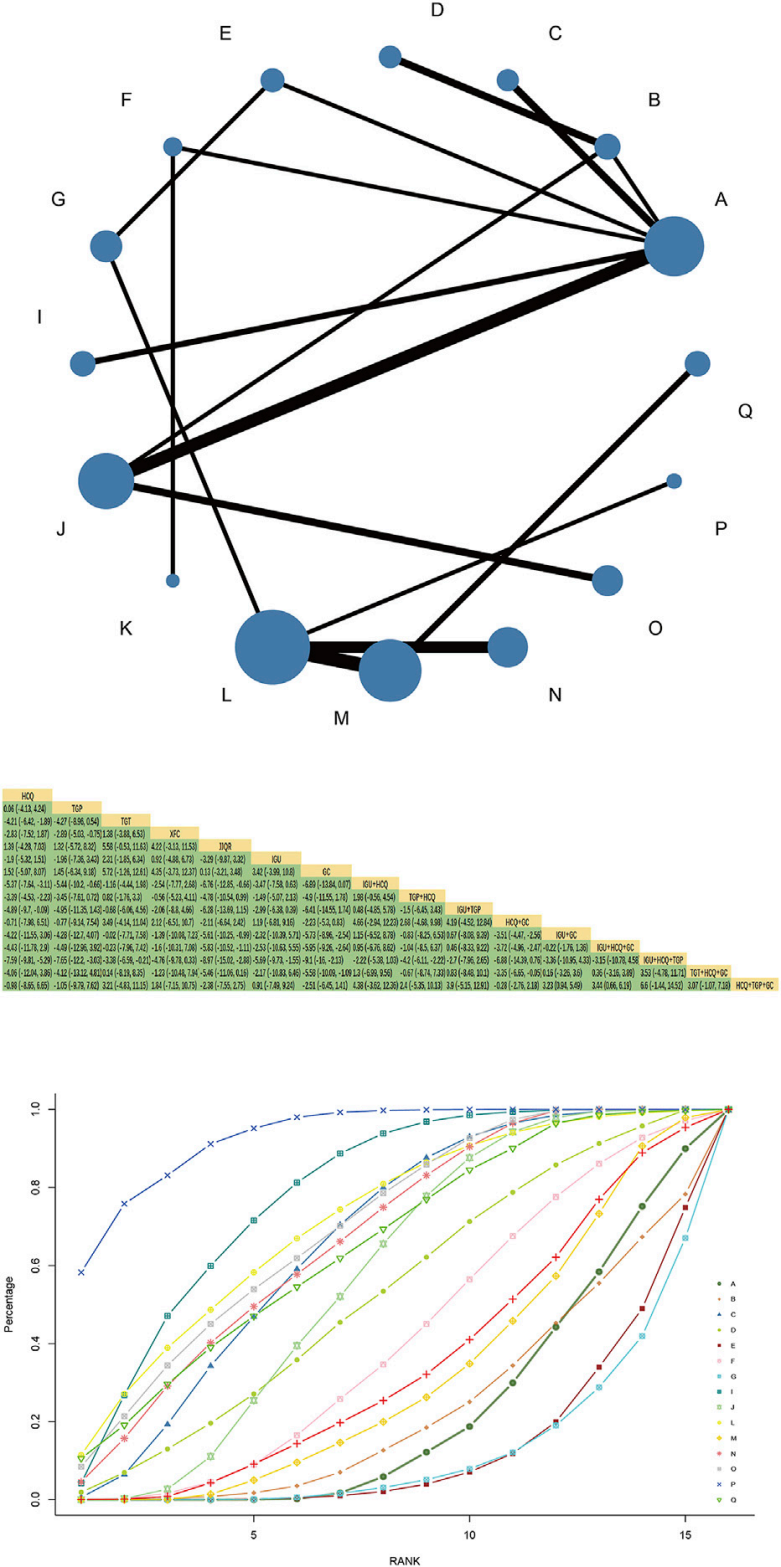
IgG network diagram, league diagram, SUCRA diagram.

Forty-six RCTs reported IgG, involving 16 interventions, 4 Chinese patent medicines, and 3,438 patients, forming one closed loop; HCQ and TGP combined with HCQ had the largest node and sample size. TGP + HCQvsHCQ was the most studied with the thickest line segment. The results of the network Meta-analysis showed that TGT was superior to HCQ [MD = −4.21, 95%CI= (−6.42, −1.89)], XFC was superior to TGP [MD = −2.89, 95%CI= (−5.03, −0.75)] under single treatment measure. TGP, XFC, and JJQR were not significantly different from HCQ and IGU (*p* > 0.05). TGP combined with HCQ was superior to HCQ alone [MD = −3.39, 95%CI= (−4.53, −2.23)] (Figure 4).

#### 3.5.2 SUCRA probability ranking

SUCRA probability values are ranked as:IGU + HCQ + TGP(SUCRA = 96.0)>IGU + HCQ + GC(SUCRA = 76.2)>IGU + GC(SUCRA = 69.4)>IGU + HCQ (SUCRA = 68.0)>TGP + HCQ (SUCRA = 67.7)>TGP + TGT (SUCRA = 65.6)>IGU + TGP(SUCRA = 57.4)> TGT (SUCRA = 53.7) = XFC(SUCRA = 53.7)>HCQ + GC(SUCRA = 42.7)>HCQ + TGP + GC.

(SUCRA = 42.1)>IGU(SUCRA = 33.6)>TGP(SUCRA = 24.2)>HCQ (SUCRA = 20.9)>GC(SUCRA = 18.6)>JJQR (SUCRA = 17.2).

### 3.6 Schirmer trial

#### 3.6.1 Evidence network and network meta-analysis

**FIGURE 5:**
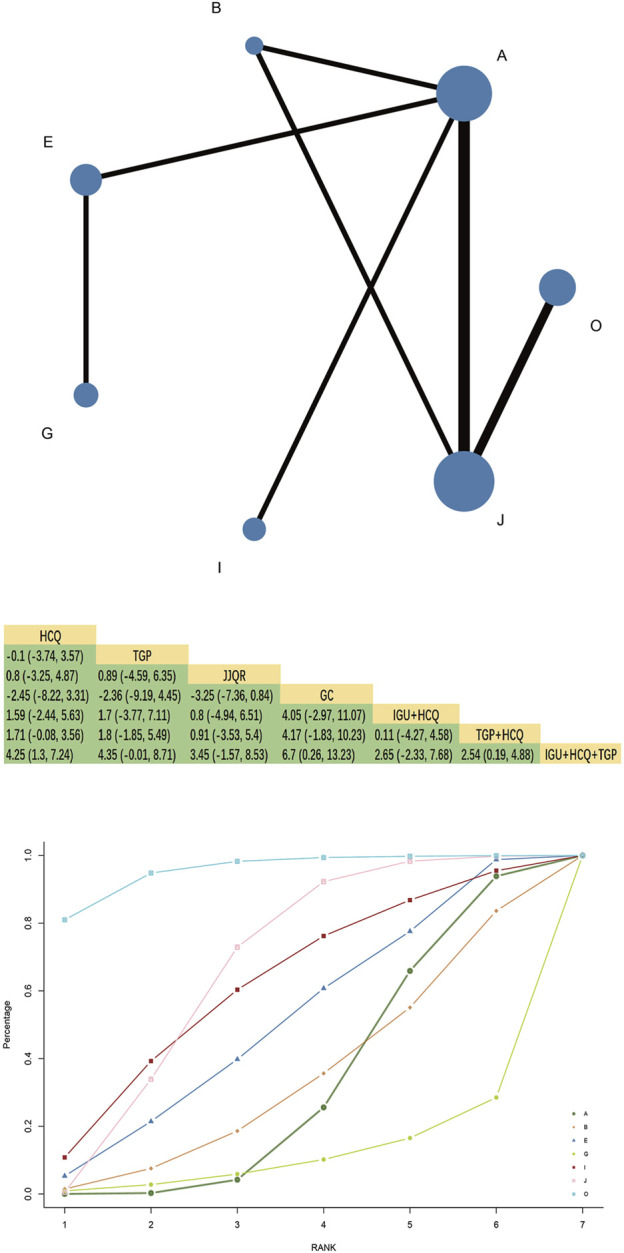
Schirmer trial network diagram, league diagram, SUCRA diagram.

The Schirmer trial was reported in 18 RCTs, involving seven interventions, 2 Chinese patent medicines, and 1,387 patients, forming one closed loop; HCQ and TGP combined with HCQ had the largest node and sample size. TGP + HCQ vs. HCQ and IGU + TGP + HCQ vs. TGP + HCQ had the most studies and the thickest line segments. The network Meta-analysis results showed that the confidence intervals included 0 compared to a single treatment measure, suggesting no significant difference in improving Schirmer between Chinese patent medicine and Western medicine alone (*p* > 0.05). IGU + HCQ + TGP was superior to TGP + HCQ [MD = 2.54, 95%CI= (0.19, 4.88), P< 0.05] and HCQ alone [MD = 4.25, 95%CI= (1.30, 7.24), P< 0.05] (Figure 5).

#### 3.6.2 SUCRA probability ranking

SUCRA probability values are ranked as:IGU + HCQ + TGP(SUCRA = 95.6)>TGP + HCQ (SUCRA = 66.2)>IGU + HCQ (SUCRA = 61.5)>JJQR (SUCRA = 50.6)>TGP(SUCRA = 33.7)>HCQ (SUCRA = 31.6)> GC(SUCRA = 10.7).

### 3.7 Salivary flow rate

#### 3.7.1 Evidence network and network meta-analysis

**FIGURE 6:**
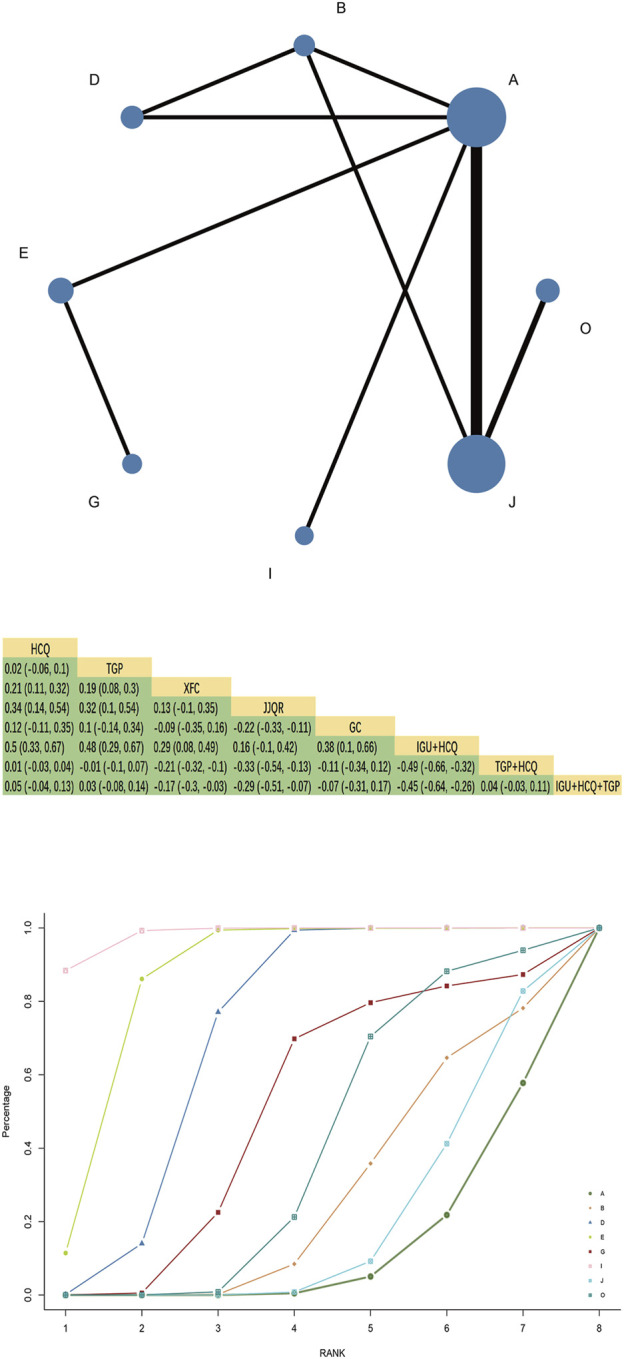
Salivary flow rate network diagram, league diagram, SUCRA diagram.

Twenty-one RCTs reported a salivary flow rate involving eight interventions, 3 Chinese patent medicines, 1,642 patients, and forming two closed loops. HCQ and TGP combined with HCQ had the largest node and sample size. TGP + HCQ vs. HCQ had the most studies and the thickest line segments. The results of the network Meta-analysis showed that JJQR [MD = 0.34, 95%CI= (0.14, 0.54), P< 0.05] and XFC were superior to HCQ [MD = 0.21, 95%CI= (0.11, 0.32), P< 0.05], and TGP had the same efficacy as HCQ. There was no statistically significant difference [MD = 0.02, 95%CI= (−0.06, 0.1), P> 0.05] (Figure 6).

#### 3.7.2 SUCRA probability ranking

SUCRA probability values are ranked as:IGU + HCQ (SUCRA = 98.2)>JJQR (SUCRA = 85.3)>XFC(SUCRA = 70.0)>GC(SUCRA = 49.2)>IGU + HCQ + TGP(SUCRA = 39.2)>TGP(SUCRA = 26.7)>TGP + HCQ (SUCRA = 19.2)>HCQ (SUCRA = 12.2).

### 3.8 Total effective rate

#### 3.8.1 Evidence network and network meta-analysis

**FIGURE 7:**
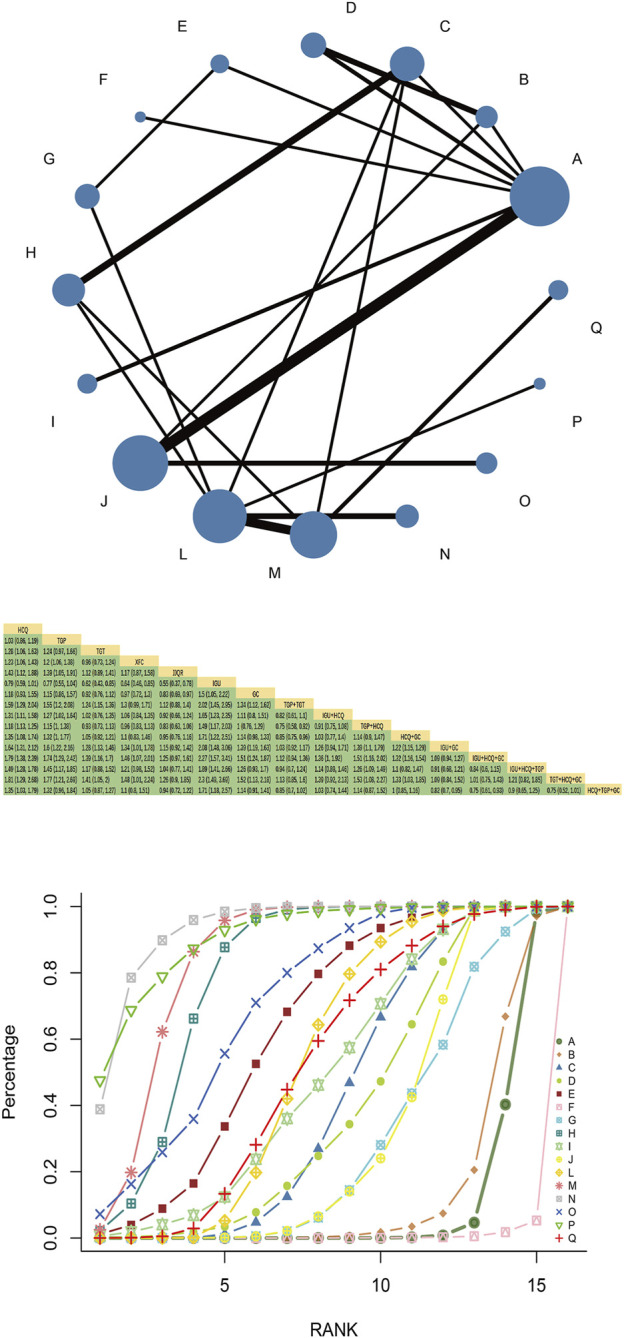
Total effective rate network diagram, league diagram, SUCRA diagram.

Fifty-four RCTs reported total response rates involving 16 interventions, 4 Chinese patent medicines, and 4,337 patients. Multiple closed loops were formed between the interventions. HCQ had the largest node and the largest sample size. TGP + HCQvsHCQ was the most studied with the thickest line segment. The results of the network Meta-analysis showed that TGT, XFC, and JJQR were superior to HCQ and IGU (*p* < 0.05), and there was no significant difference between TGP and HCQ or IGU (*p* > 0.05). TGP combined with HCQ was superior to HCQ alone [MD = 1.18, 95%CI= (1.13, 1.25)] (Figure 7).

#### 3.8.2 SUCRA probability ranking

SUCRA probability values are ranked as: IGU + HCQ + GC (SUCRA = 93.6 > TGT + HCQ + GC (SUCRA = 91.0) > IGU + GC (SUCRA = 84.2 > TGP + TGT (SUCRA = 79.8)> IGU + HCQ + TGP (SUCRA = 71.1 > JJQR (SUCRA = 63.1 > HCQ + GC (SUCRA = 53.2 > HCQ + TGP + GC (SUCRA = 51.6 > IGU + HCQ (SUCRA = 48.3 > TGT (SUCRA = 43.0 > XFC (SUCRA = 38.4 > TGP + HCQ (SUCRA = 30.6 > GC (SUCRA = 28.8)> TGP (SUCRA = 12.8 > HCQ (SUCRA = 9.5 > IGU (SUCRA = 0.6).

### 3.9 Adverse events

Forty-two RCTs reported adverse events, as detailed in Supplementary Table S3. Four studies had no apparent discomfort, and 38 reported gastrointestinal discomfort, abnormal liver function, leukopenia, blurred vision, rash, and itching. Still, there was no dropout due to adverse events.

### 3.10 Consistency analysis

Bayesian P values generated by the node-splitting method were used to verify consistency between direct and indirect comparisons (Supplementary Figure S1). All P values exceeded 0.05, indicating a satisfactory level of consistency.

### 3.11 Publication bias

**FIGURE 8:**
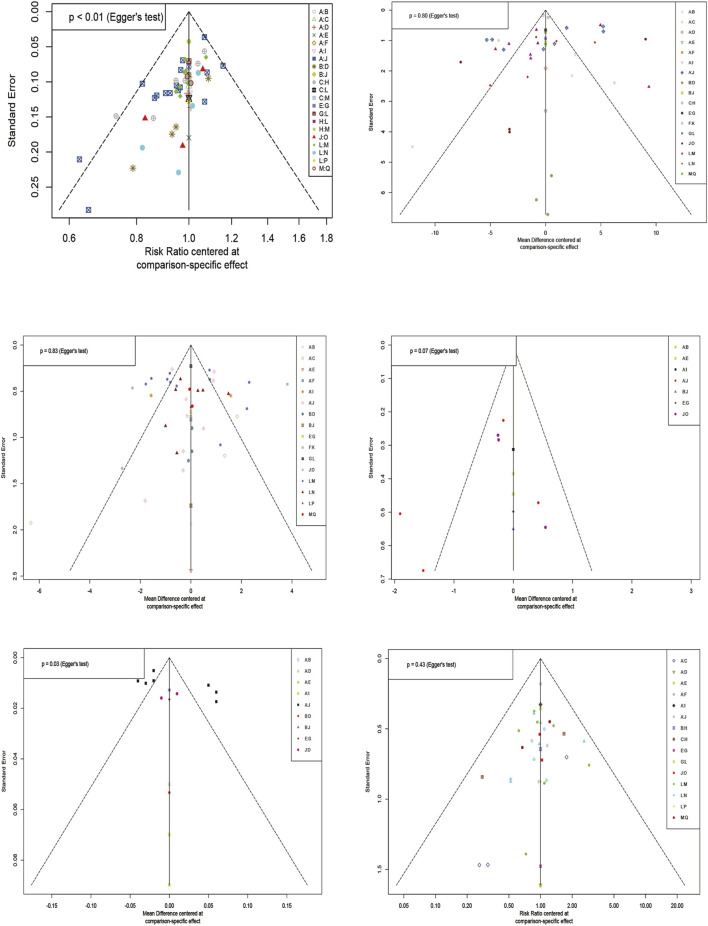
Funnel plot of each outcome indicator.

The results of the comparative-corrected funnel plot showed that most of the included literature was symmetrically distributed around the zero line. However, there was still a tiny part of the discrete distribution, indicating that there may be a certain degree of publication bias and a small sample effect (Figure 8).

## 4 Discussion

Western medicine treatments for SS usually rely on the extrapolation of the therapeutic effects of other autoimmune diseases, which are less selective. The treatment and management of patients often rely on experience, and there is a lack of evidence of treatment effects (Seror et al., 2021). With the gradual introduction of TCM, TCM is more and more widely used in the treatment of Sjogren’s syndrome. Natural drugs such as traditional Chinese medicine have strong pharmacological activities, anti-inflammatory and immune regulation effects.

In this study, various traditional Chinese medicines, such as TGT, TGP, radix scrophulariae, ophiopogon japonicus can be used to treat SS. A total of 66 RCTs were included in this study, which related 4 Chinese patent medicines, 3 Western medicines, and five outcome indicators, and achieved direct and indirect comparisons between different interventions used alone or in combination, and initially filled the academic gap of priority comparison of Chinese patent medicines commonly used in clinical treatment of SS. Of these 17 drug therapies, 4 were proprietary Chinese medicines, 3 were Western medicines (2 DMARDs and hormones), and 10 were different combinations of proprietary Chinese and Western medicines. The results of the study showed that IGU + HCQ + TGP tended to be recommended as the best treatment when the three drugs were used in combination because it ranked the highest in ESR (96.0%), IgG (96.0%), and Schirmer test (95.6%), and the risk of adverse events was relatively low. When the two drugs are combined, IGU + GC and TGT + TGP are good choices for reducing ESR and IgG. Although TGP + HCQ vs. HCQ had the most studies, TGP combined with HCQ ranked relatively low in each outcome indicator when the two drugs were compared. When a drug is used alone, TGT or XFC is better in reducing ESR or IgG, while JJQR alleviates dry mouth-eye, and improves Schirmer trial and Salivary flow rate.

However, some TCM will inevitably cause certain damage to the heart, liver, kidney, stomach and other organs, as well as common adverse reactions, such as blood system damage, gastrointestinal reaction, liver and kidney damage, skin itching, headache, constipation, amenorrhea,etc.For example, studies have shown that TGT has some reproductive toxicity (Zhang et al., 2023),which may lead to the risk of amenorrhea in female patients with Sjogren’s syndrome.

Tripterygium wilfordii polyglycosides are the components extracted from the root of the Eualaceae plant Tripterygium wilfordii. It has the effects of eliminating wind and dampness, reducing swelling, and relieving pain. It is widely used in autoimmune diseases due to its potent anti-inflammatory and immunomodulatory effects. Consistent with the results of this study, a study (Liu J. et al., 2022) showed that TGT could alleviate inflammatory response and improve symptoms such as dry mouth and blood viscosity in SS model mice. Total paeony glucosides derived from the dried root of Paeonia lactiflora in the Ranunculaceae family are the most studied Chinese patent medicine for SS. Hanying Mei (Mei et al., 2021) found that total glucosides of paeony can reduce the inflammatory response in mice with Sjogren’s syndrome by regulating the activity of TLR4/MyD88/NF-κB signaling pathway and play an anti-inflammatory role. Both have been recommended by the “Guidelines for the Diagnosis and Treatment of Sjogren’s Syndrome based on TCM Syndromes” (Jiang et al., 2024). The results of this study also showed that the combined IGU + HCQ + TGP regimen was significantly effective in reducing ESR, IgG and improving Schirmer trial, ranking first, but there is no study explaining the synergistic mechanism of TGP on IGU + HCQ.Desiccation affects more than 95% of patients with SS (Brito-Zerón et al., 2016). JJQR has a good effect on improving salivary flow, perhaps because its ingredients contain ginseng, ophiopogon and radix scrophulariae, which have the effect of nourishing qi and Yin, generating fluid and quenching thirst.

## 5 Limitation

There have been few indirect clinical studies on the treatment of SS by different TCMs combined with CWM; therefore, the differences in the therapeutic effects of different TCMs combined with CWM are not clear. In this study, network meta-analysis was used to clearly compare the efficacy of different TCMs combined with CWM to guide clinical treatment and provide certain suggestions and aid. However, there are still several shortcomings in this study. First,We found that not all studies specified the randomization process, which may have a particular publication bias; Second, Considering the large number of included studies, the differences between studies may affect the applicability of the network meta-analysis (transitivity assumption). Although all the studies we included were randomized controlled trials, there may still be significant inter-study differences in randomization methods, sample size settings, and intervention protocols. Additionally, the basic characteristics of the study populations are also important factors influencing the transitivity assumption. In the studies we included, the population’s age and disease duration were around 50 years and 5 years, respectively. We believe there are no significant differences in these two population characteristics between studies, thus meeting the transitivity assumption. However, some potential population characteristics, such as gender ratio and ethnicity, may still exhibit inter-study differences that could impact the robustness of our conclusions. Therefore, our conclusions will be interpreted and considered with caution. Next, some treatments had few papers, which may lead to statistical bias. Finally, TCMs are not widely used in other countries. Therefore, almost all selected papers in this NMA were from China, which may have caused regional, language,and racial biases. We hope that in the future there will be large-scale RCTs in different countries to further provide more reliable data.

## 6 Conclusion

Through the network meta-analysis concluded that TCMs combined with CWM had more significant clinical efficacy and safety in treating SS compared to only CWM, and also obtained the order of optimal interventions for different outcome measures. Among them, IGU + HCQ + TGP may be the best intervention. TGP + HCQ, TGP + TGT, IGU + TGP can be considered as an alternative to IGU + HCQ when reducing ESR and IgG. TGT and XFG decrease ESR and IgG with good clinical effects. JJQR may have an advantageous role in relieving xerostomia and dry eyes. The aim of the results of this study is to provide some advice and help for clinical application.

## Data Availability

The original contributions presented in the study are included in the article/Supplementary Material, further inquiries can be directed to the corresponding authors.
