# Supplementary material for: Network meta-analysis of integrated traditional Chinese and Western medicine in the treatment of Sjogren’s syndrome
Source: Front Pharmacol. 2024 Nov 27;15:1455969. doi: 10.3389/fphar.2024.1455969 (PMC11631580; doi:10.3389/fphar.2024.1455969)
Supplement: Supplementary file 1 [file DataSheet1.pdf]

Supplementary Material

Network meta-analysis of integrated traditional Chinese and Western medicine in the treatment of Sjogren's syndrome

Xieli Ma[1†], Zixia Liu [1†], Tian Chang[1†], Chuanhui Yao[1†], Yuchen Yang[1], Biyue Shang[1], Jiameng Liu[1], Congmin Xia[1], Xun Gong[1*], Quan Jiang[1*]

[1]Department of Rheumatology, Guang'anmen Hospital, China Academy of Chinese Medical Sciences,China

*** Correspondence:**
Xun Gong：gongxun8269@163.com；Quan Jiang：doctorjq@126.com

1 TABLE

1.1 Supplementary Table S1| PubMed search strategy

Databases:	PubMed	
Search number	Search query	Numbers
#1	（（"Sjogren's Syndrome" [Mesh]）OR（（（（"Syndrome, Sjogren's" [Title/Abstract]）OR（"Sjogren Syndrome" [Title/Abstract]））OR（"Sicca Syndrome" [Title/Abstract]））OR（"Syndrome, Sicca" [Title/Abstract]）））	17046
#2	（（（（（（（（（（（（（（（"tripterygium glycosides" [Title/Abstract]）OR（"Total glucoside capsules of Paeony" [Title/Abstract]））OR（"Pavlin capsules" [Title/Abstract]））OR（"Xinfeng capsule" [Title/Abstract]））OR （"Methotrexate" [Title/Abstract]））OR（"hydroxychloroquine" [Title/Abstract]））OR（"hydroxychloroquine sulfate" [Title/Abstract]））OR（"leflunomide" [Title/Abstract]））OR（"Iguratimod" [Title/Abstract]））OR（"thalidomide" [Title/Abstract]））OR（"hormone" [Title/Abstract]））OR（"methylprednisolone" [Title/Abstract]））OR（"metacortandracin" [Title/Abstract]））OR（"prednisone" [Title/Abstract]））OR（"hydrocortisone" [Title/Abstract]））	580956
#3	#1 AND #2	583

1.2 Supplementary Table S2| SUCRA ranking in the network graph of each outcome indicator

Intervention	ESR	IgG	Schirmer trial	Salivary flow rate	Total effective rate
A(HCQ)	14(SUCRA=20.9)	13(SUCRA=20.9)	6(SUCRA=31.6)	8(SUCRA=12.2)	15(SUCRA=9.5)
B(TGP)	13(SUCRA=24.2)	12(SUCRA=24.2)	5(SUCRA=33.7)	6(SUCRA=26.7)	14(SUCRA=12.8)
C(TGT)	8(SUCRA=53.7)	8(SUCRA=53.7)	-	-	10(SUCRA=43.0)
D(XFC)	9(SUCRA=46.4)	8(SUCRA=53.7)	-	3(SUCRA=70.0)	11(SUCRA=38.4)
E(JJQR)	16(SUCRA=17.3)	15(SUCRA=17.2)	4(SUCRA=50.6)	2(SUCRA=85.3)	6(SUCRA=63.1)
F(IGU)	12(SUCRA=33.6)	11(SUCRA=33.6)	-	-	16(SUCRA=0.6)
G(GC)	15(SUCRA=18.7)	14(SUCRA=20.9)	7(SUCRA=10.7)	4(SUCRA=49.2)	13(SUCRA=28.8)
H(TGP+TGT)	6(SUCRA=65.5)	6(SUCRA=65.6)	-	-	4(SUCRA=79.8)
I(IGU+HCQ)	4(SUCRA=67.9)	4(SUCRA=68.0)	3(SUCRA=61.5)	1(SUCRA=98.2)	9(SUCRA=48.3)
J(TGP+HCQ)	5(SUCRA=67.6)	5(SUCRA=67.7)	2(SUCRA=66.2)	7(SUCRA=19.2)	12(SUCRA=30.6)
K(IGU+TGP)	7(SUCRA=57.4)	7(SUCRA=57.4)	-	-	-
L(HCQ+GC)	10(SUCRA=42.8)	9(SUCRA=42.7)	-	-	7(SUCRA=53.2)
M(IGU+GC)	3(SUCRA=69.5)	3(SUCRA=69.4)	-	-	3(SUCRA=84.2)
N(IGU+HCQ+GC)	2(SUCRA=76.4)	2(SUCRA=76.2)	-	-	1(SUCRA=93.6)
O(IGU+HCQ+TGP)	1(SUCRA=96.0)	1(SUCRA=96.0)	1(SUCRA=95.6)	5(SUCRA=39.2)	5(SUCRA=71.1)
P(TGT+HCQ+GC)	-	-	-	-	2(SUCRA=91.0)
Q(HCQ+TGP+GC)	11(SUCRA=42.2)	10(SUCRA=42.1)	-	-	8(SUCRA=51.6)

Note:Numbers 1-16 indicate the SUCRA ranking number of the interventions in the outcome indicators of this column, and the smaller the number, the larger the SUCRA value, the higher the ranking.

1.3 Supplementary TABLE S3 | Adverse events to the included articles.

study	Intervention	Adverse reaction	
		T	C
Wang et al., 2013	XFC/HCQ	-	-
Zhu et al., 2016	XFC/HCQ	1 of ①;	None
Ma, 2012	TGT /HCQ	2 of ③，2 of ②	1 of ③，1 of ②，1 of ④
Guo et al., 2012	TGT /HCQ	1 of ③，1 of ②	None
Ma et al., 2021	TGT /HCQ	1 of ③，1 of ②，1 of ⑥	None
Zhao, 2020	IGU/HCQ	2 of ①，1 of ②，1 of ⑤	1 of ①，1 of ②，2 of ⑤，1 of ③
Fan et al., 2015	XFC/TGP	-	-
Wang et al., 2014	XFC/TGP	-	-
Shao, 2016	XFC/TGP	-	-
Yang et al., 2011	XFC/TGP	-	-
Zhang et al., 2011	JJQR/HCQ	None	1 of ②
Zhang et al., 2009	JJQR	None	2 of ②，1 of ⑩
Liu et al., 2020	TGP+ TGT / TGT	-	-
Ye et al., 2019	TGP+ TGT / TGT	-	-
Wu et al., 2017	TGP+ TGT / TGT	-	-
Wang, 2017	TGP+ TGT / TGT	4 of ①	2 of ①

Zhao, 2019	TGP+ TGT / TGT	3 of ①，1 of ⑧	6 of ①，6 of ⑧
Gan et al., 2022	TGT +TGP/TGP	4 of ①，2 of ⑨	4 of ①
Lu et al., 2021	IGU+HCQ/HCQ	3 of ①，2 of ②，7 of ⑤，1 of ⑦	2 of ①，2 of ②，9 of ⑤，1 of ⑦
Ji et al., 2019	IGU+HCQ/HCQ	-	-
Zhao, 2018	TGP+HCQ/HCQ	5 of ①	4 of ①
Chen et al., 2017	TGP+HCQ/HCQ	None	None
Gao, 2021	TGP+HCQ/HCQ	-	-
Shi et al., 2023	TGP+HCQ/HCQ	2 of ②，2 of ①，2 of ⑤，1 of ⑦	1 of ④，1 of ①，3⑤
He, 2010	TGP+HCQ/HCQ	3 of ④：④ of ①	1 of ④；1 of ①
Li et al., 2016	TGP+HCQ/HCQ	-	-
Wang, 2019	TGP+HCQ/HCQ	1 of ④，4 of ①	2 of ④，1 of ⑦，1 of ①
Tang et al., 2020	TGP+HCQ/HCQ	-	-
Zhang, 2015	TGP+HCQ/HCQ	Three of④①	Two of④①
Yin, 2011	TGP+HCQ/HCQ	1 of ④，3 of ①	2 of ④
Liu et al., 2022	TGP+HCQ/HCQ	-	-
Lu et al., 2019	TGP+HCQ/HCQ	7 of ①，1 of ⑤	6 of ①
Zhao, 2023	TGP+HCQ/HCQ	-	-
Xu et al., 2022	TGP+HCQ/HCQ	None	None
Chu, 2021	TGP+HCQ/HCQ	-	-
Li, 2019	IGU+TGP/IGU	-	-
Zhao et al., 2013	TGP+HCQ/HCQ/TGP	-	-
Feng et al., 2021	HCQ+GC/GC	7 of ①，6 of ⑤，1 of ②，2 of ⑦	5 of ①，4 of ⑤，3 of ②
Zhang, 2019	IGU+GC/HCQ+GC	-	-
Zhang et al., 2019	IGU+GC/HCQ+GC	4①，1 of ⑤，1 of ⑤，1 of ③	3 of ①，2 of ②，1 of ⑤
Xu et al., 2017	IGU+GC/HCQ+GC	5 of ①，1 of ②，3 of ⑤，1 of ③	4 of ①，3 of ②，5 of ⑤
Zhao, 2019	IGU+GC/HCQ+GC	2 of ③	3 of ③
Yu, 2020	IGU+GC/HCQ+GC	-	-
Gu, 2020	IGU+GC/HCQ+GC	1 of ①， 1 of ⑤	2 of ①，1 of ②，2 of ⑤，2 of ③
Jiang et al., 2016	IGU+GC/HCQ+GC	1 of ③	1 of ⑤
Gu, 2022	IGU+GC/HCQ+GC	1 of ①， 1 of ⑤	2 of ①， 1 of ⑤
Xia et al., 2017	IGU+GC/HCQ+GC	-	-
Wang et al., 2020	IGU+GC/HCQ+GC	None	None
Wang, 2018	IGU+GC/HCQ+GC	-	-
Luo et al., 2018	IGU+GC/HCQ+GC	3 of ①，1 of ②，3 of ⑤	2 of ①，2 of ②，4 of ⑤，1 of ③
Li et al., 2022	IGU+GC/HCQ+GC	3 of ①， 2 of ⑤，1 of ③	8 of ①， 2 of ⑤，1 of ③
Liu et al., 2023	IGU+GC/HCQ+TGP+GC	6 of ①，1 of ②，3 of ⑤	4 of ①，1 of ②，2 of ⑤，1 of ③
Liu, 2022	IGU+GC/HCQ+TGP+GC	2 of ①， 2 of ⑤	2 of ①，2 of ②，1 of ⑤
Ding et al., 2022	IGU+HCQ+GC/HCQ+GC	1 of ①，1 of ②， 1 of ③	1 of ①，1 of ⑤
Jiang et al., 2014	IGU+HCQ+GC/HCQ+GC	3 of ①，1 of ②	2 of ①，2 of ②，1 of ⑤
Li et al., 2020	IGU+HCQ+GC/HCQ+GC	1 of ①，1 of ②，1 of ⑤	4 of ①，3 of ②，3 of ⑤

Meng et al., 2023	IGU+HCQ+GC/HCQ+GC	2 of ①，1 of ②	2 of ①
Rao et al., 2022	IGU+HCQ+GC/HCQ+GC	-	-
Jia, 2020	IGU+HCQ+GC/HCQ+GC	3 of ①，1 of ②，3 of ⑤	2 of ①，2 of ②，4 of ⑤，1 of ③
Luo et al., 2019	IGU+HCQ+GC/HCQ+GC	无	无
Li et al., 2018	IGU+HCQ+GC/HCQ+GC	2 of ①，1 of ②，6 of ⑤	4 of ①，1 of ②，5 of ⑤
Wang et al., 2019	IGU+HCQ+TGP/HCQ+TGP	1 of ①，1 of ⑤，1 of ③	2 of ①，1 of ⑤
Zhang, 2021	IGU+HCQ+TGP/HCQ+TGP	-	-
Xie et al., 2020	IGU+HCQ+TGP/HCQ+TGP	7 of ①	4 of ①，1 of ②
Chen et al., 2022	IGU+HCQ+TGP/HCQ+TGP	-	-
Ju et al., 2022	TGT +HCQ+GC/HCQ+GC	1 of ①，1 of ④，1 of ②	1 of ①，1 of ④

Note: ① Gastrointestinal reaction; ② Abnormal liver function; ③ Abnormal blood routine; ④ Blurred vision; ⑤ itching rash; ⑥ abnormal menstruation; ⑦ Dizziness;⑧cardiovascular abnormalities; ⑨ fatigue; ⑩ Increased blood glucose.

2 FIGURE

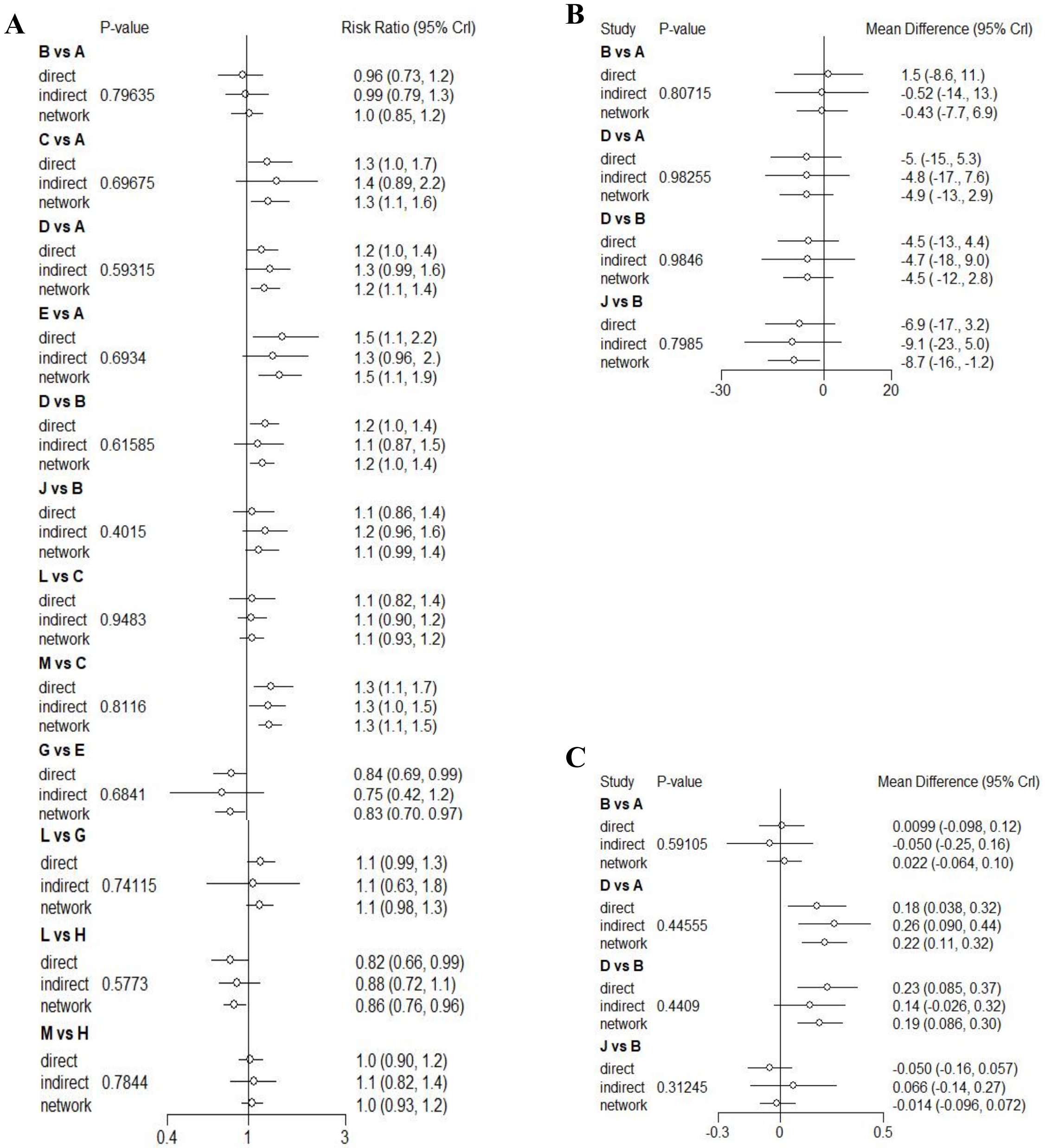

2.1 FIGURE S1.Forest plot of consistency analysis;A:Total effective rate;B:ESR;C:Total effective rate.
